# Making an impact: the Journal of Foot and Ankle Research

**DOI:** 10.1186/1757-1146-5-16

**Published:** 2012-07-09

**Authors:** Hylton B Menz, Mike J Potter, Alan M Borthwick, Shannon E Munteanu, Karl B Landorf

**Affiliations:** 1 Lower Extremity and Gait Studies Program, Faculty of Health Sciences, La Trobe University, Bundoora, VIC, Australia; 2 Faculty of Health Sciences, University of Southampton, Southampton, United Kingdom; 3 Department of Podiatry, Faculty of Health Sciences, La Trobe University, Bundoora, VIC, Australia

## 

"*“When you can measure what you are speaking about, and express it in numbers, you know something about it; but when you cannot measure it, when you cannot express it in numbers, your knowledge is of a meagre and unsatisfactory kind”*"

Lord Kelvin, 1883 [1].

Science is all about quantification. It is therefore somewhat ironic that the mechanism by which science is disseminated – academic publishing – has yet to develop an acceptable way of measuring itself. Several fundamental issues regarding the scholarly publishing enterprise remain largely unresolved, such as how journal performance should be assessed, how journals should be compared and ranked, and how the impact of individual manuscripts should be evaluated. These issues are of relevance not only to journal editors and publishers, but also to librarians, who need to decide which journals to purchase, to researchers, who need to decide which journals to select for submission to, and to clinicians, who need to decide which journals they should read.

By far the most widely used measure of journal performance is the Impact Factor, first developed by Eugene Garfield in 1955 as a means of selecting which journals to include in the *Science Citations Index*[2-4]. The Impact Factor represents the average number of citations received per paper published in that journal during the two preceding years. For example, the Impact Factor of a journal in 2011 is calculated as follows:

A = the number of times articles published in 2009 and 2010 were cited by indexed journals during 2011.

B = the total number of "citable items" published by that journal in 2009 and 2010. ("citable items" include articles, reviews or proceedings and excludes editorials and letters).

2011 Impact Factor = A/B.

Due largely to its simplicity, the Impact Factor has been widely adopted as a measure of journal “prestige”, and many researchers consider the Impact Factor when selecting a journal to which to submit their work. Impact Factors have also been used to assess the output of researchers seeking academic promotion [5] and to guide research resource allocation [6], although these broader applications of the Impact Factor in isolation are not recommended by Thomson Reuters, the company that owns the Impact Factor and publishes journal rankings through the *Journal Citation Reports* database [7].

Several valid criticisms have been made of the Impact Factor. These criticisms relate primarily to: (i) the questionable assumption that citation rates are a valid measure of research impact or quality, (ii) the entirely arbitrary (and for some disciplines, very short) window of two years in which citations to a manuscript “count” toward the Impact Factor calculation, (iii) the skewed distribution of manuscript citations within a journal (i.e. very highly cited papers make a disproportionate contribution to a journal’s Impact Factor), and (iv) limitations in the coverage of journals in the Thomson ISI database, which has an English-language and US bias [8-11]. There is also evidence of editors intentionally manipulating journal content to achieve a higher Impact Factor by instructing authors to cite more papers within the journal, and by publishing review papers and commentaries with high levels of self-citation [12-14].

Despite these significant limitations, it would appear that the Impact Factor will continue to play a role in journal rankings until a more appropriate metric takes its place, therefore it would be disingenuous of the editorial team at *JFAR* to suggest that the publication of our first Impact Factor is not of considerable interest to us. *JFAR* was formally accepted for tracking by Thomson ISI on November 18, 2011, and our first Impact Factor was therefore calculated from 2009–2011 data. In 2009–2010, *JFAR* published 66 manuscripts, which attracted 88 citations in 2011. Therefore, our first Impact Factor is 88/66 = 1.333. In other words, on average, each manuscript published in *JFAR* in 2009–2010 attracted an average of 1.333 citations in 2011.

The obvious question that arises from this is whether 1.333 is a “good” Impact Factor, however the answer to this question depends largely on the frame of reference. Our journal is clearly not going to give the *New England Journal of Medicine* (Impact Factor = 53.298) a run for its money. However, relative to our competitors, *JFAR*’s Impact Factor compares extremely well. Of the 14 English-language journals specifically focused on foot and ankle research, only five are tracked by Thomson ISI (see Table 1), and *JFAR*’s Impact Factor is the highest in this group of journals. In a broader context, *JFAR* is listed under Thomson ISI’s Orthopaedics category, and is ranked 32^nd^ out of the 63 journals in this discipline.

**Table 1:** 2011 Impact Factors of English language foot-specific journals

**Journal**	ISSN*	**Impact Factor**
** *Journal of Foot and Ankle Research* **	**1757-1146**	**1.333**
*Foot and Ankle International*	1071-1007	1.218
*Foot and Ankle Clinics*	1083-7515	0.709
*Journal of the American Podiatric Medical Association*	8750-7315	0.567
*Journal of Foot and Ankle Surgery*	1067-2516	0.516
*The Foot*	0958-2592	-
*Foot and Ankle Online Journal*	1941-6806	-
*Foot and Ankle Specialist*	1938-7636	-
*Foot and Ankle Surgery*	1268-7731	-
*The Diabetic Foot*	1462-2041	-
*Foot and Ankle Quarterly*	1068-3100	-
*Journal of Diabetic Foot Complications*	2160-7036	-
*Clinics in Podiatric Medicine and Surgery*	1559-6486	-
*Techniques in Foot and Ankle Surgery*	15360644	-
*Diabetic Foot and Ankle*	2000-625X	-

* International standard serial number.

**Figure 1:**
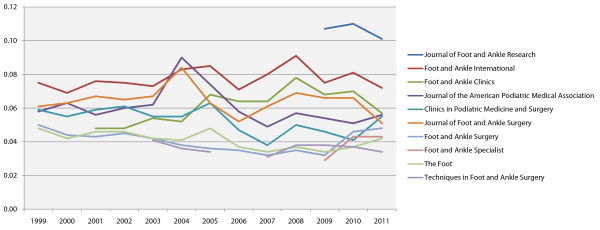
SCImago Journal Rankings of English language foot-specific journals, 1999–2011.

Although the Impact Factor is the most well-known journal performance metric, an alternative, freely accessible journal ranking known as the SCImago Journal Rank has recently been developed by the technology company SCImago Lab [15]. The SCImago Journal Rank uses Elsevier’s more extensive SCOPUS database rather than Thomson ISI’s Web of Science, and uses a more complex algorithm similar to Google’s PageRank which accounts for both the number of citations received by a journal and the importance or prestige of the journals where the citations came from. The SCImago Journal Rank shares many of the limitations of the Impact Factor, however its developers argue that it is a more accurate reflection of a journal’s prestige due to: (i) the broader journal coverage of the SCOPUS database, (ii) a wider citation “window” of three years, (iii) a correction factor to prevent excessive self-citation (the proportion of self-citations is restricted to 33% of the total), and (iv) consideration of the “quality” of citations [16]. Of the 14 English-language journals specifically focused on foot and ankle research, ten are tracked by SCOPUS and therefore have a SCImago Journal Rank (see Table 2). Since 2009 when SCOPUS tracking commenced, *JFAR*’s SCImago Journal Rank has consistently been the highest in this group of journals (see Figure 1), and it is currently ranked 29^th^ out of the 143 journals listed in the Orthopaedics and Sports Medicine category [17].

**Table 2:** 2011 SCImago Journal Rankings of English-language foot-specific journals

**Journal**	ISSN*	**SCImago Journal Rank**
** *Journal of Foot and Ankle Research* **	**1757-1146**	**0.101**
*Foot and Ankle International*	1071-1007	0.072
*Foot and Ankle Clinics*	1083-7515	0.057
*Journal of the American Podiatric Medical Association*	8750-7315	0.056
*Clinics in Podiatric Medicine and Surgery*	1559-6486	0.055
*Journal of Foot and Ankle Surgery*	1067-2516	0.051
*Foot and Ankle Surgery*	1268-7731	0.048
*Foot and Ankle Specialist*	1938-7636	0.043
*The Foot*	0958-2592	0.042
*Techniques in Foot and Ankle Surgery*	15360644	0.034
*Foot and Ankle Online Journal*	1941-6806	-
*The Diabetic Foot*	1462-2041	-
*Foot and Ankle Quarterly*	1068-3100	-
*Journal of Diabetic Foot Complications*	2160-7036	-
*Diabetic Foot and Ankle*	2000-625X	-

* International standard serial number.

Notwithstanding our concerns regarding the validity of both the Impact Factor and SCImago Journal Rank as measures of journal prestige, the editors of *JFAR* are very satisfied with the journal’s initial rankings, particularly given that we only commenced publication in July 2008. The publication of our Impact Factor and SCImago Journal Rank will undoubtedly influence the journal selection process of researchers in our discipline, so it is likely that the overall number of submissions to *JFAR* will increase in future years. However, rather than agonising over ubiquitous yet flawed journal performance metrics, we will continue to make editorial decisions based on the relevance and scientific quality of individual manuscripts, keeping in mind the (frequently cited) words of Nobel prize winning biologist Sydney Brenner: “Before we develop a pseudoscience of citation analysis, we should remind ourselves that what matters absolutely is the scientific content of a paper and that nothing will substitute for either knowing it or reading it” [18].
